# Nucleophagy in *Aspergillus oryzae* is Mediated by Autophagosome Formation and Vacuole-Mediated Degradation

**DOI:** 10.1007/s00284-024-03838-y

**Published:** 2024-08-20

**Authors:** Mau Hashimoto, Satoshi Kimura, Manabu Arioka

**Affiliations:** 1 Department of Biotechnology, The University of Tokyo, 1-1-1 Yayoi, Bunkyo-Ku, Tokyo, 113-8657 Japan; 2 Electron Microscope Section, Technology Advancement Center, Graduate School of Agricultural and Life Sciences, The University of Tokyo, 1-1-1 Yayoi, Bunkyo-Ku, Tokyo, 113-8657 Japan; 3 Collaborative Research Institute for Innovative Microbiology (CRIIM), The University of Tokyo, 1-1-1 Yayoi, Bunkyo-Ku, Tokyo, 113-8657 Japan

## Abstract

We previously reported autophagy-mediated degradation of nuclei, nucleophagy, in the filamentous fungus *Aspergillus oryzae*. In this study, we examined whether nuclei are degraded as a whole. We generated *A. oryzae* mutants deleted for orthologs of *Saccharomyces cerevisiae YPT7* and *ATG15* which are required, respectively, for autophagosome-vacuole fusion and vacuolar degradation of autophagic bodies. Degradation of histone H2B-EGFP under starvation conditions was greatly decreased in the Δ*Aoypt7* and Δ*Aoatg15* mutants. Fluorescence and electron microscopic observations showed that autophagosomes and autophagic bodies surrounding the entire nuclei were accumulated in the cytoplasm of Δ*Aoypt7* and the vacuole of Δ*Aoatg15*, respectively. These results indicate that nuclei are engulfed in the autophagosomes as a whole and transported/released into the vacuolar lumen where they are degraded.

**Supplementary Information:**

The online version contains supplementary material available at 10.1007/s00284-024-03838-y.

## Introduction

Autophagy is a starvation-induced process in which cytoplasmic components are delivered to vacuoles/lysosomes for degradation. Through autophagy, cells cope with starvation by recycling and reusing the degradation products such as amino acids. Autophagy also plays roles in the removal of abnormal proteins and damaged organelles [1], elimination of intracellularly invaded bacteria [2], and cell development and differentiation [3]. In macroautophagy, a common type of autophagy, autophagy-related proteins (Atg proteins) first assemble to form an initial structure called pre-autophagosomal structure (PAS) [4]. Next, Atg proteins and other related factors work together to form a double-membrane vesicle called phagophore, which extends to surround the target for degradation, forming a spherical double-membrane structure called autophagosome. Subsequently, the autophagosome is transported to and fuses with the vacuole/lysosome, releasing the structures called autophagic bodies derived from the inner membrane of autophagosome. The autophagic bodies are then degraded through the lipase activity present in the vacuole/lysosome, and the degradation target is finally exposed to vacuolar hydrolytic enzymes to undergo degradation [5].

Among macroautophagy, the mechanism in which specific organelles or proteins are degraded is called selective autophagy, and nucleophagy is a type of selective autophagy in which nuclei are degraded [6]. Several types of nucleophagy are known. In piecemeal microautophagy of the nucleus, the specific interaction of Nvj1 in the nuclear envelope and Vac8 located in the vacuolar membrane triggers the invagination of small blebs that contain a part of *Saccharomyces cerevisiae* nucleus into the vacuole. These blebs are eventually pinched off and released into the vacuolar lumen where they are degraded by vacuolar hydrolases. Macroautophagic degradation of nuclei also occurs in *S. cerevisiae* in which Atg39 plays a role as a receptor for the nucleus. Atg39 spans the outer nuclear membrane (ONM) or surrounding ER and associates with the inner nuclear membrane (INM) through its amphipathic helices. The core Atg proteins are recruited through Atg39 binding to Atg11, and subsequently to Atg8, leading to autophagosome formation. Finally, double-membrane vesicles whose outer and inner membranes are derived from ONM and INM, respectively, are surrounded by autophagosomes with nucleoplasm in their lumen [7, 8]. In addition, there is also a mechanism called NPC-phagy in which the nuclear pore complex (NPC) is degraded. NPC is a huge protein assembly consisting of more than 500 proteins, including approximately 30 types of nucleoporins, and is essential for the transport of various components between the nucleus and cytoplasm. Upon nitrogen source starvation or inhibition of the target of rapamycin activity, NPC becomes surrounded by autophagosomes and undergoes degradation [9].

Using the filamentous fungus *Aspergillus oryzae*, we previously reported a unique phenomenon in which the entire nucleus is incorporated by a giant autophagosome and degraded by autophagy [10]. Although this degradation was thought to be mediated by the macroautophagy pathway, the specific degradation pathway has not been investigated. To confirm whether nuclear degradation by nucleophagy in *A*. *oryzae* follows the macroautophagic processes, i.e., formation of autophagosome, fusion of autophagosome with vacuole, and degradation of autophagic body in the vacuole, in this study, we analyzed the nucleophagy in the mutants deleted for *Aoatg1*, *Aoatg8*, *Aoypt7*, and *Aoatg15* which are orthologs for *S. cerevisiae ATG1*, *ATG8*, *YPT7*, and *ATG15*, respectively. Atg1 is a kinase essential for the initiation of autophagy, and Atg8 is an ubiquitin-like protein required for membrane fusion and phagophore expansion during autophagosome formation [11]. Ypt7 is a Rab GTPase with its active form localizing to the vacuolar membrane and interacting with a tethering complex called HOPS (homotypic vacuole fusion and protein sorting). The HOPS complex also interacts with SNARE (soluble *N*-ethylmaleimide-sensitive factor attachment protein receptor) proteins such as Ykt6 on autophagosomes, and Vam3, Vit1, and Vam7 on the vacuolar membrane [12, 13]. Through these interactions, Ypt7 plays an essential role for the fusion of autophagosomes and vacuoles [12, 14]. Atg15 is a vacuolar phospholipase that degrades the membrane of autophagic bodies, and in its absence, autophagic bodies accumulate in the vacuolar lumen [15, 16]. Analysis of these disruptants verified that nuclei are engulfed in the autophagosomes and transported to/released into the vacuolar lumen where they are degraded.

## Material and methods

### Strains and growth media

The *A. oryzae* strains used in this study are listed in Table S1. *A. oryzae* wild-type strain RIB40 [17] was used as a DNA donor, and the adenine auxotrophic mutant NSRku70-1-1 because of the mutation in the *adeA* gene [18] was used to disrupt the *Aoypt7* gene using the *adeA* marker. NSRku70-1-1 transformed by *adeA* (NSRku70-1-1A) [19] was used as a control strain for the phenotypic analyses. M medium [0.2% NH_4_Cl, 0.1% (NH_4_)_2_SO_4_, 0.05% KCl, 0.05% NaCl, 0.1% KH_2_PO_4_, 0.05% MgSO_4_·7H2O, 0.002%, FeSO_4_·7H_2_O, and 2% glucose (pH 5.5)] supplemented with 0.15% methionine (M + met) was used as a selective medium for disrupting the *Aoypt7* gene. Czapek-Dox (CD) medium [0.3% NaNO_3_, 0.2% KCl, 0.1% KH_2_PO_4_, 0.05% MgSO_4_·7H_2_O, 0.002% FeSO_4_·7H_2_O, and 2% glucose (pH 5.5)] supplemented with 0.0015% methionine (CD + met) was used as a selective medium for identifying positive clones of *Aoypt7* and *Aoatg15* disruptants expressing EGFP-AoAtg8 and *Aspergillus nidulans* histone H2B (AnH2B)-EGFP. CD medium including 1% casamino acids (CD + CA) and CD lacking sodium nitrate (CD-N) or glucose (CD-C) were used for induction of autophagy. Dextrin–polypeptone–yeast extract (DPY) medium was used for pre-culture in each experiment. Potato dextrose (PD; Nissui, Tokyo, Japan) agar medium was used for phenotypic analysis.

### Construction of Aoypt7 disruptant

The plasmid pUC19_Aoypt7 deletion was constructed by the following method to disrupt the *Aoypt7* gene. The 1.0 kb upstream region of the *Aoypt7* gene, the 1.0 kb downstream region of the *Aoypt7* gene containing the *Sma* I recognition site (written in small letters in the sequence below), and *adeA* gene with *Not* I recognition site (small letters) were amplified by PCR using the following primer pairs, which contained overlapping sequence (underlined): pUC19_Aoypt7_up_Fw (5′-CTCGGTACCCGGGGATCGCTGCGGCGTGAGTCCTGTGA-3′) and pUC19_Aoypt7_up_Rv (5′-GTCTAGCACCCATGCGGCCGGCTGTGGCTATGTAAAGA-3′), and pUC19_Aoypt7_down_Fw (5′-AGCTCGGTACCCGGGGATCgctagcTACCCTAGTGATGGACATG-3′) and pUC19_Aoypt7_down_Rv (5′-AGGTCGACTCTAGAGGATCATCGTCTGGGACGTAGGTC-3′), pUC19_adeA_Fw (5′-GCTCGGTACCCGGGGATCgcggccgcATGGGTGCTAGACTCACAT-3′) and pUC19_adeA_Rv (5′-CCATCACTAGGGTAGCTAGCTAGACCGCAGGAACCTTA-3′), respectively (Table S2). First, the amplified downstream fragment was introduced into the pUC19 plasmid at the *Bam*H I site, then the *adeA* gene into the *Sma* I site, and finally the upstream fragment into the *Not* I site in this order using In-Fusion HD Cloning Kit (Takara). Using the plasmid pUC19_Aoypt7 deletion as a template, the sequence containing the deletion cassette, which consisted of the upstream region of *Aoypt7* (1.0 kb), *adeA* gene (2.8 kb) and downstream region of *Aoypt7* (1.0 kb), was amplified by PCR using the primers pUC19_Aoypt7_up_Fw and pUC19_Aoypt7_down_Rv and was then transformed into *A*. *oryzae* NSRku70-1-1. Disruption of the *Aoypt7* gene was confirmed by PCR with the primers Aoypt7 check 500-F (5′-TCCTCTGTCCTCTCCTGAGA-3′) and Aoypt7 check 500-R (5′-TACGGTCGTGTATTGCAGGC-3′).

### Construction of strains expressing EGFP-AoAtg8 or AnH2B-EGFP

The plasmid pgEGA8 containing *A*. *oryzae niaD* gene as a selection marker and *egfp* gene linked to the *Aoatg8* [20] was used to transform the *Aoypt7* and *Aoatg15* disruptants. The plasmid pNH2BG containing *A. oryzae niaD* gene and *egfp* linked to *A*. *nidulans histone h2b* gene [21] was used to transform the *Aoatg1*, *Aoypt7,* and *Aoatg15* disruptants.

### Western blotting

The mycelia were frozen in liquid nitrogen and disrupted using ShakeMan6 (Bio Medical Science). Extraction buffer [50 mM Tris–HCl (pH 7.5), 1 mM PMSF, Protease Inhibitor Cocktail (Promega)] was added to the disrupted mycelia, and the suspension was centrifuged at 13,000 rpm for 8 min. The supernatant was recovered, and mixed with 5 × Laemmli Sample Buffer [165 mM Tris–HCl (pH 6.8), 7.5% SDS, 50% glycerol, 0.00125% BPB, 5% 2-mercaptoethanol], and boiled for 3 min at 100 °C. Samples were separated by SDS–PAGE, and analyzed by immunoblotting using antibodies against GFP (Clontech, 632380) and Alexa Fluor® 680 AffiniPure™ goat anti-mouse IgG (H + L) (Jackson). The EGFP bands were detected by using LI-COR Odyssey Imaging System, and the band intensities were analyzed using ImageJ. To calculate the degradation ratio of EGFP-fused proteins (%), the band intensity of free EGFP was divided by the sum of intensities of free EGFP and EGFP-fused full-length protein using ImageJ.

### Fluorescence microscopic observation

Conidia or hyphae were cultured in a glass-based dish (VIOLAMO, 4-2673-01) in 100 µL CD + CA medium for 24 h at 30 °C. The media were then replaced with fresh CD + CA, CD-N, or CD-C medium, and the mycelia were further incubated for 6 h at 30 °C. The cells were then fixed in 4% paraformaldehyde, stained by 4’,6-diamidino-2-phenylindole (DAPI) at 2.5 µg/ml, and observed using BZ-X700 microscope (Keyence) equipped with light-emitting diode light sources and the excitation/emission filter sets for DAPI (340–380 nm/435–485 nm) and EGFP (450–490 nm/500–550 nm), respectively. The experiments were performed multiple times to ensure reproducibility.

### Transmission electron microscopic observation

Cells were fixed with 2.5% glutaraldehyde, 4% paraformaldehyde, and 50 mM phosphate buffer (pH 7.2) at 4 °C overnight, and postfixed with 1% osmium tetroxide, 50 mM phosphate buffer for 3 h at room temperature. Fixed specimens were dehydrated in a graded ethanol series, substituted in propylene oxide, and embedded in Spurr Low-Viscosity Embedding Media (Polyscience, USA), followed by polymerization at 70 °C for 16 h. Ultrathin sectioning (80 nm thickness) was done with a diamond knife (Diatome, USA) and an Ultracut UCT ultramicrotome (Leica, Germany). Sections were picked up on Formvar-coated copper grids, stained with aqueous uranyl acetate and lead citrate, and examined under a JEM-1400 Plus (Jeol, Japan) operated at 100 kV. The experiments were performed multiple times to ensure reproducibility. *Accession numbers *AoYpt7: XP_001824054.

## Results

### Identification and functional analysis of Aoypt7 in A. oryzae

Identification of *Aoatg1*, *Aoatg8*, and *Aoatg15* in *A. oryzae* has been reported [20, 22, 23]. To identify Ypt7 ortholog in *A. oryzae*, we searched the protein database and found a sequence, named hereafter AoYpt7 (XP_001824054), displaying 68% amino acid sequence identity to Ypt7 (Fig. S1). The binding regions for the HOPS complex and guanine nucleotides in Ypt7 were conserved. To analyze the function of AoYpt7 in autophagy, we obtained a strain in which the *Aoypt7* gene was disrupted by homologous recombination using the *adeA* selection marker (Fig. S2). The phenotype of Δ*Aoypt7* was observed after 4 days of culture on PD medium (Fig. S3a). Δ*Aoypt7* exhibited a reduced growth rate and markedly decreased formation of conidia compared to the wild type (Fig. S3b). These phenotypes were consistent with those commonly observed in the strains deficient for autophagy function [20, 22, 23], suggesting that AoYpt7 also plays an essential role in autophagy.

**Fig. 1:**
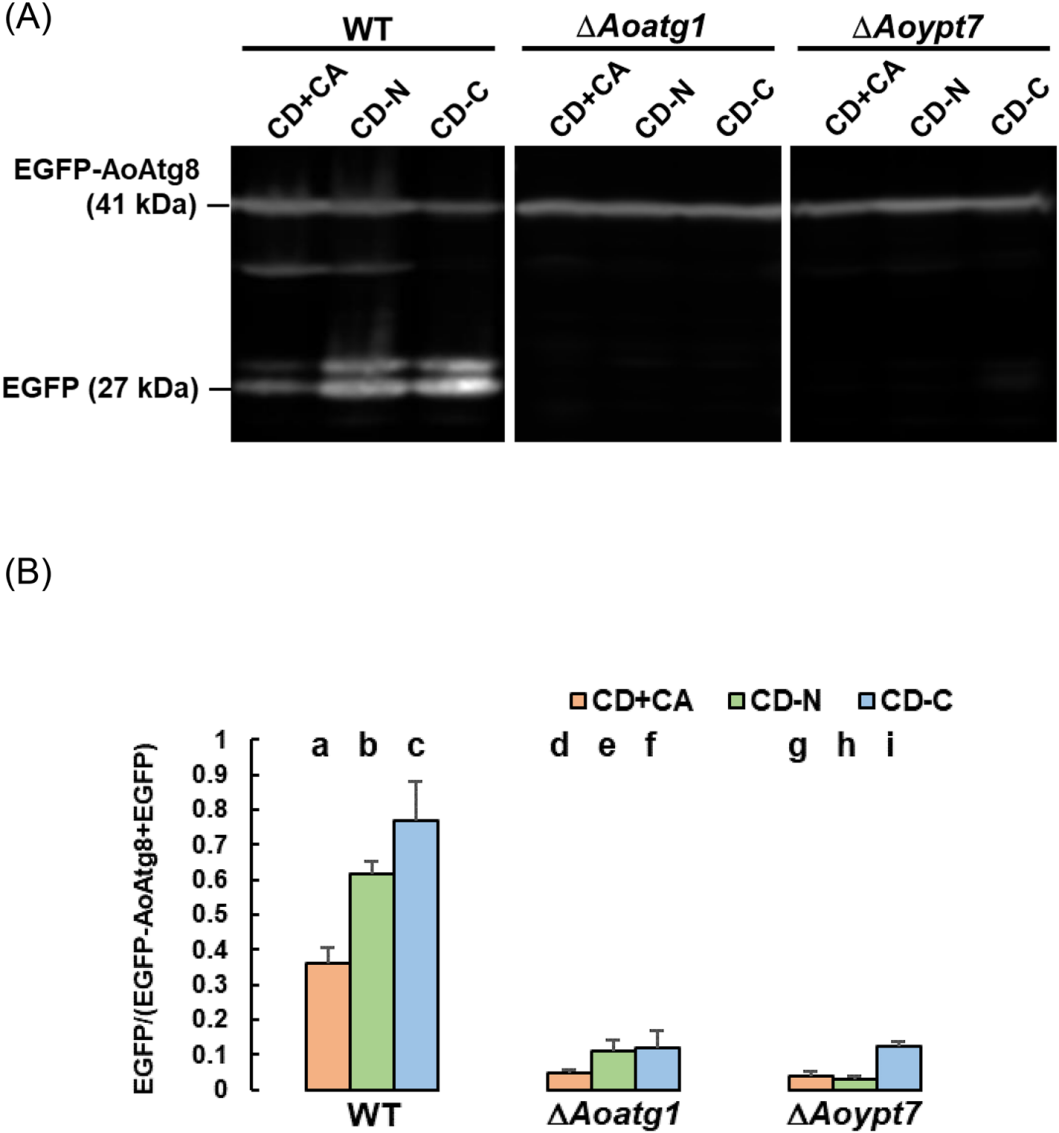
EGFP-AoAtg8 processing assay. **a** Wild type (PA8GAtg8), Δ*Aoatg1* (DA1EA8), and Δ*Aoypt7* (DAoypt7AoAtg8) strains were inoculated into DPY liquid medium and incubated at 30 °C for 24 h. Cells were then transferred to CD + CA (1% casamino acid), CD-N, or CD-C medium, and incubated at 30 °C for 6 h. The mycelia were then frozen in liquid nitrogen, disrupted, and proteins were extracted. Western blotting was performed using an anti-EGFP antibody. **b** Fluorescence intensities of the detected bands were quantified by ImageJ, and the degradation ratio (EGFP/(EGFP-AoAtg8 + EGFP)) was calculated. To quantify the degree of degradation, only the fluorescent intensity of the band for free EGFP (27 kDa) in (**a**) was measured as an indicator of complete degradation of EGFP-AoAtg8. Error bars, standard deviation (*n* = 3). Statistical difference by *t* test was detected between the following pairs: a–b, a–c, *p* < 0.01; a–d, a–g, b–h, c–f, c–i, *p* < 0.001; b–e, *p* < 0.0001

To examine the general autophagy activity of Δ*Aoypt7*, we performed the EGFP-AoAtg8 processing assay [24, 25]. Processing assay allows the quantification of autophagic degradation of target proteins in the vacuole. When the target protein tagged with EGFP is degraded through autophagy, only the EGFP moiety accumulates in the vacuole since EGFP is resistant to vacuolar proteases. The ratio of free EGFP to the total EGFP intensity calculated by Western blotting indicates the degradation ratio of the target protein. Since Atg8 is localized on autophagosomes and is degraded in the vacuole through autophagy, GFP-Atg8 has been used as a marker for quantifying the general autophagy activity in the processing assay [24]. In *A*. *oryzae*, EGFP-AoAtg8 has also been used in the same way [25]. After culturing EGFP-AoAtg8-expressing strain in the nutrient-rich DPY medium for 24 h at 30 °C, they were transferred to a synthetic medium containing 1% casamino acids (CD + CA), nitrogen-starved medium (CD-N), or carbon-starved medium (CD-C) for another 6 h. The AoAtg8 degradation ratio in the wild-type strain was about 35% in CD + CA but increased to 60% and 75% in CD-N and CD-C, respectively (Fig. 1a, b). To confirm that the degradation of EGFP-AoAtg8 was mediated by autophagy, this assay was also performed in the *Aoatg1* disruptant. In the Δ*Aoatg1* strain, the degradation ratio of EGFP-AoAtg8 significantly decreased to around 10% in all conditions. This verifies that starvation-induced degradation of EGFP-AoAtg8 was mediated by autophagy. In the Δ*Aoypt7* strain, the degradation ratios were comparable to those in the Δ*Aoatg1* strain, indicating that AoYpt7 is essential for the general autophagy activity.

### Nuclear degradation requires genes involved in the macroautophagy pathway

**Fig. 2:**
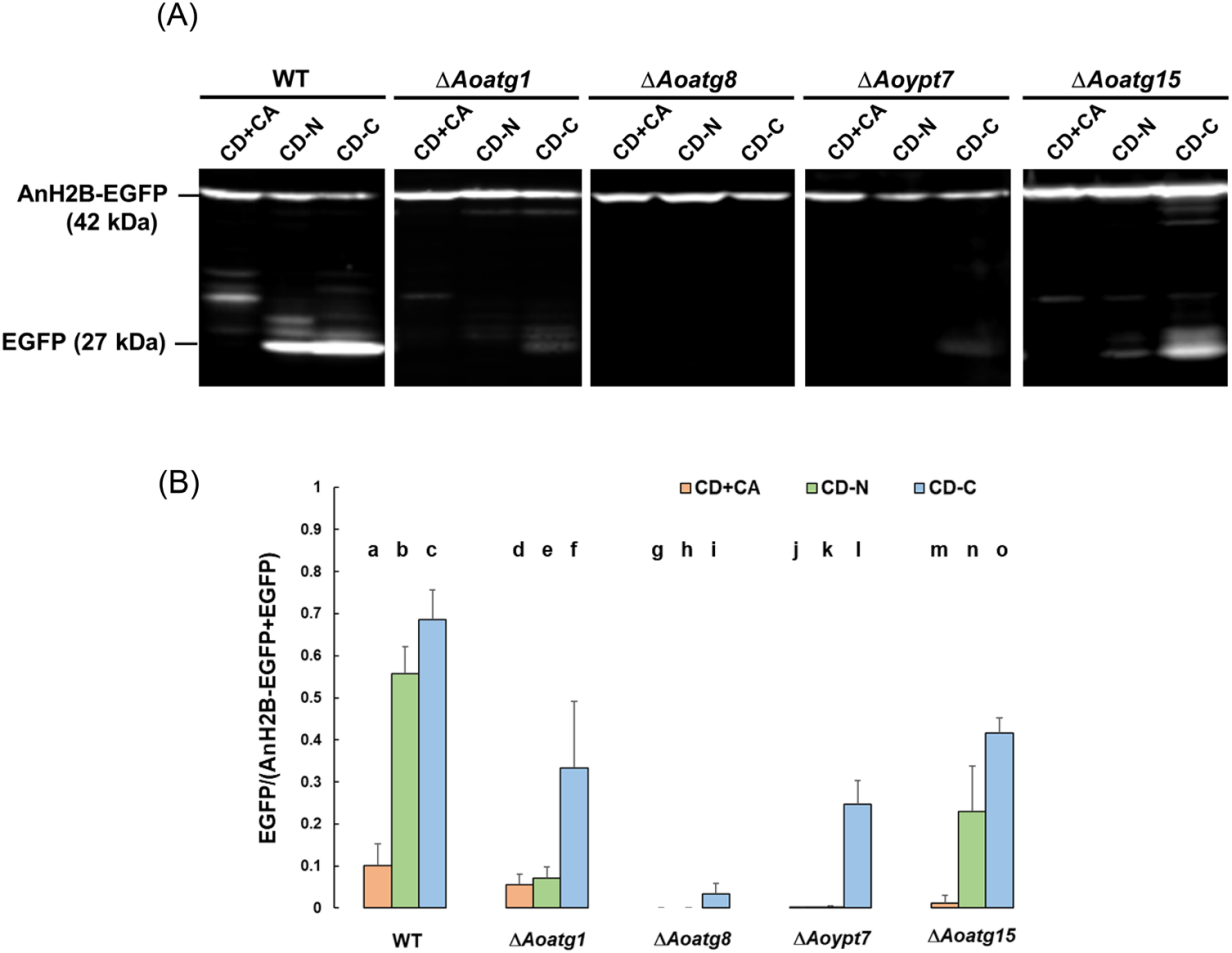
AnH2B-EGFP processing assay. **a** Wild-type strain (NSRku70-1-1A-AnH2B-EGFP), Δ*Aoatg1* strain (DAoatg1AnH2B), Δ*Aoatg8* strain (PA8GAtg8), Δ*Aoypt7* strain (DAoypt7AnH2B), and Δ*Aoatg15* strain (Aoatg15-AnH2B-EGFP) were inoculated into DPY liquid medium and incubated at 30 °C for 24 h. Then, shifted to CD + CA (1% casamino acid) medium, CD-N medium, and CD-C medium, and incubated at 30 °C for 6 h. The mycelia were then frozen in liquid nitrogen, disrupted, and proteins were extracted. Western blotting was performed using the anti-EGFP antibody. **b** The fluorescence intensity of the detected bands was quantified by ImageJ, and the degradation ratio (EGFP/(AnH2B-EGFP + EGFP)) was calculated. To quantify the degree of degradation, only the fluorescent intensity of the band for free EGFP (27 kDa) in (**a**) was measured as an indicator of complete degradation of AnH2B-EGFP. Error bars, standard deviation (WT, n = 6; Δ*Aoatg8*, Δ*Aoypt7*, Δ*Aoatg15*, n = 3; Δ*Aoatg1*, *n* = 5). Statistical difference by *t* test was detected between the following pairs: b–n, *p* < 0.05; a–b, c–f, c–l, *p* < 0.01; a–c, b–k, b–q, c–l, *p* < 0.001; b–e, *p* < 0.0001

To examine whether the degradation of nuclei requires genes involved in the macroautophagy pathway, we analyzed nucleophagy activity in the disruptants of *Aoatg1*, *Aoatg8*, *Aoypt7*, and *Aoatg15*. Since *Aspergillus nidulans* histone H2B (AnH2B), when expressed in *A. oryzae*, was shown to localize to the nucleus [21], AnH2B-EGFP processing assay has been used to quantify nucleophagy activity in *A. oryzae* [26]. AnH2B-EGFP-expressing strain was cultured, and autophagy was induced in the same way as the EGFP-AoAtg8 processing assay. In the wild-type strain, the degradation ratio in CD + CA was about 10%, while it was significantly increased to about 55% and 65% in CD-N and CD-C, respectively (Fig. 2a, b). The degradation of AnH2B-EGFP in the Δ*Aoatg1*, Δ*Aoatg8*, and Δ*Aoypt7* strains in the starvation conditions significantly decreased, indicating that AnH2B-EGFP was degraded through autophagy upon nutrient starvation. It is of note, however that in the carbon-starved condition, compared to fully abolished degradation of AnH2B-EGFP in Δ*Aoatg8*, in Δ*Aoatg1* and Δ*Aoypt7,* a certain level of degradation occurred, suggesting that autophagy-independent degradation of AnH2B-EGFP might also occur. In the Δ*Aoatg15* strain, starvation-induced degradation of AnH2B-EGFP also decreased, but a significant level of degradation still occurred, indicating that the autophagy-deficient phenotype of Δ*Aoatg15* was somewhat leaky. Overall, these results indicate that nuclei are degraded through autophagy.

### The entire nucleus is surrounded by autophagosomes and transported to the vacuole

**Fig. 3:**
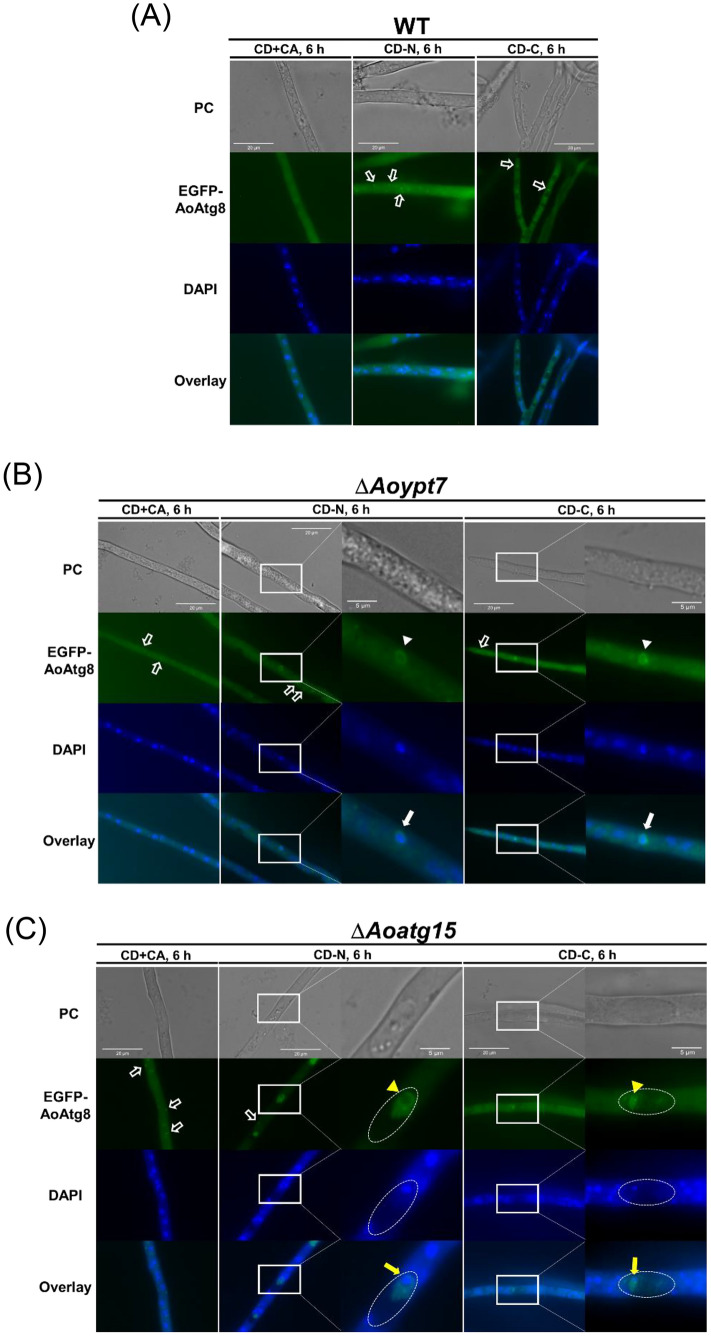
Fluorescence microscopy of EGFP-AoAtg8-expressing strains. Wild type (PA8GAtg8; (**a**)), Δ*Aoypt7* (DAoypt7; (**b**)), and Δ*Aoatg15* (**c**) strains were grown in DPY liquid medium for 24 h at 30 °C. Cells were then shifted to CD + CA (1% casamino acid), CD-N, or CD-C medium and incubated for 6 h at 30 °C. They were fixed with 4% paraformaldehyde, and the nuclei were stained with DAPI. PC, phase contrast. White open arrows, PAS; white arrowheads, autophagosomes; white arrows, autophagosomes surrounding the entire nucleus; dotted circles, vacuoles; yellow arrowheads, autophagic bodies; yellow arrows, the entire nucleus is surrounded by autophagic bodies. Bars = 20 µm, 5 µm (zoom) (Color figure online)

To analyze the mode of transport of nuclei into vacuoles, we observed the EGFP-AoAtg8-expressing strains which have been used for labeling autophagosomes by fluorescence microscopy [23, 27] (Fig. 3). To efficiently observe autophagosomes and autophagic bodies, it is desirable to inhibit autophagy after their formation and before their degradation. Therefore, in addition to the wild-type strain, we used the Δ*Aoypt7* strain in which the fusion of vacuoles and autophagosomes is inhibited, and the Δ*Aoatg15* strain in which the degradation of autophagic bodies is blocked. These strains were pre-cultured in the DPY medium for 24 h, then shifted to CD + CA, CD-N, or CD-C, and incubated for 6 h at 30 °C. Nuclei were stained with DAPI before observation. In the wild-type strain, multiple green puncta (indicated by open arrows in Fig. 3a) were observed in the cytoplasm in CD-N and CD-C, but not in CD + CA. These were assumed to be PAS, the initial structure of assembly of autophagy-related proteins according to the previous studies [20, 28]. Vacuoles were not well developed and, therefore, not clearly observed in these growth conditions. In Δ*Aoypt7*, PAS-like structures were observed under all conditions including CD + CA (open arrows in Fig. 3b). In addition, green fluorescent ring-like structures (arrowheads) which were presumed to be autophagosomes were observed in the cytoplasm in CD-N and CD-C. Importantly, these structures surrounded the DAPI-stained entire nucleus (white arrows) as reported in our previous study [10]. In Δ*Aoatg15*, in addition to the PAS-like structures observed in the cytoplasm as in Δ*Aoypt7*, multiple green fluorescent ring-like structures (yellow arrowheads in Fig. 3c) were observed in the vacuoles. These were likely to be autophagic bodies, and in some cases, the entire nucleus was surrounded by these autophagic bodies (yellow arrows). Collectively, these data strongly suggest that when the cells were starved, the entire nucleus was first surrounded by autophagosome, and then released into the vacuolar lumen enclosed in the autophagic body upon fusion of autophagosome with the vacuole.

**Fig. 4:**
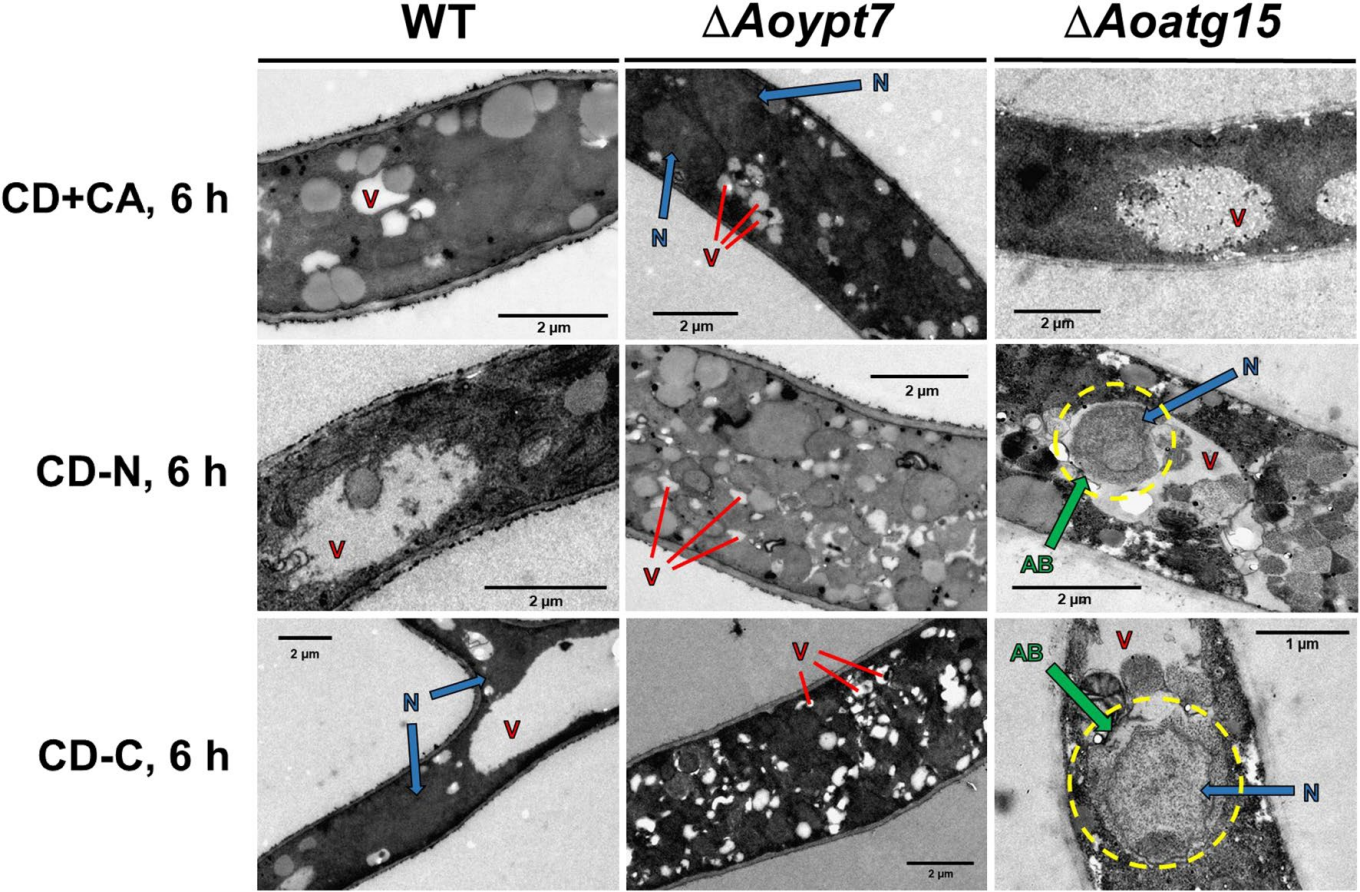
Observation using transmission electron microscopy. After inoculation of wild-type strain (NSRku70-1-1A), Δ*Aoypt7* strain (DAoypt7), and Δ*Aoatg15* strain (Δ*Aoatg15*) into DPY liquid medium and incubated at 30 °C for 24 h in a glass base dish and incubated. Then, shifted to CD + CA (1% casamino acid) medium, CD-N medium, and CD-C medium, and incubated at 30 °C for 6 h. Samples were prepared for observation and observed by transmission electron microscopy. *N* Nucleus, *V* vacuole, *AB* autophagic body; yellow-dotted circle, autophagic bodies surrounding the entire nucleus. Bars. 2 µm, 1 µm (lower right photo only)

Since autophagosomes and autophagic bodies surrounding the entire nucleus were observed by fluorescence microscopy, transmission electron microscopy was performed to observe the nuclear structures in more detail (Fig. 4). In the wild-type strain, multiple nuclei were present in the cytoplasm, and vacuoles were larger in CD-N and CD-C than in CD + CA. In Δ*Aoypt7*, highly fragmented vacuoles were observed in all conditions, and the cytoplasm was highly crowded with various membranous structures, making it difficult to observe the autophagosome-related structures. In Δ*Aoatg15*, large vacuoles were formed in CD-N and CD-C, and many autophagic bodies containing degradation targets were accumulated in the vacuole. Importantly, some autophagic bodies surrounding the entire nucleus were also observed (yellow dotted circles). These findings support the idea that in *A. oryzae* nucleophagy was a vacuole-mediated macroautophagy pathway and the nuclei are degraded as a whole.

## Discussion

Δ*Aoypt7* strain showed a reduced growth rate compared to the wild-type strain as well as an almost complete loss of ability to form conidia (Fig. S3). These phenotypes were commonly observed in autophagy-deficient strains. For example, disruption of *Aoatg8* or *Aoatg15* in *A. oryzae* also resulted in reduced growth and inhibition of the formation of aerial hyphae and conidia compared to the wild-type strain [20, 23]. In *Fusarium graminearum,* disruption of autophagy-related genes also displayed reduced growth and sporulation [29]. To verify that *Aoypt7* is required for the general autophagy activity, the degradation of EGFP-AoAtg8 was examined using the processing assay that can quantify the vacuolar degradation of target proteins (Fig. 1). In the wild-type strain, compared to non-starvation condition, the degradation of EGFP-AoAtg8 significantly increased under nitrogen and carbon starvation conditions. In contrast, as observed in Δ*Aoatg1*, in Δ*Aoypt7,* the degradation of EGFP-AoAtg8 in the starvation condition did not increase, indicating that *Aoypt7* is required for the general autophagy activity. Interestingly, in both Δ*Aoatg1* and Δ*Aoypt7*, the level of degradation of EGFP-AoAtg8 in non-starvation condition was significantly lower than that in the wild-type, suggesting that the homeostatic autophagy-mediated degradation of EGFP-AoAtg8 also occurs in non-starvation condition.

We then performed an AnH2B-EGFP processing assay to examine if nuclei are also degraded through autophagy (Fig. 2). In the wild type, the degradation of AnH2B-EGFP significantly increased in both starvation conditions compared to the non-starvation condition. In contrast, nitrogen starvation-induced degradation was abolished in Δ*Aoatg1*, Δ*Aoatg8*, and Δ*Aoypt7*, and was significantly decreased in Δ*Aoatg15*. These results indicate that nuclei are degraded through autophagy. In the carbon starvation condition, however, a significant level of degradation was observed in *Aoatg1*, *Aoypt7*, and *Aoatg15*, suggesting that autophagy-independent but AoAtg8-dependent degradation of AnH2B-EGFP might also occur. Although the precise mechanism for this degradation is not clear, one possible explanation for Δ*Aoatg15* is that in this mutant, the autophagic bodies accumulated in the vacuoles might have been disrupted during sample preparation due to the fragility of autophagic bodies in the carbon-starved condition, resulting in the exposure of AnH2B-EGFP to vacuolar proteases and its degradation. Besides, in autophagy-deficient mutants, the degradation of AnH2B-EGFP decreased even in non-starvation condition, as was observed for EGFP-AoAtg8, suggesting that nuclei are also subject to autophagy-mediated homeostatic recycling.

Fluorescence and transmission electron microscopic observations further confirmed that nuclei are degraded through autophagy (Figs. 3 and 4). Importantly, EGFP-AoAtg8-positive ring-like structures surrounding the DAPI-stained nuclei were observed in the cytoplasm of Δ*Aoypt7* grown in the starvation conditions, indicating that whole nuclei were engulfed by autophagosomes. Furthermore, in Δ*Aoatg15,* green fluorescent ring-like structures that surrounded DAPI-positive nuclei were observed in the vacuoles, suggesting that the entire nuclei contained within the autophagic bodies were accumulated in the vacuole. These observations indicate that the entire nuclei are degraded through autophagy. Although the complementation experiments were not fully performed in this study, decreased formation of conidia, the phenotype shared by autophagy-deficient strains, was restored in the complemented strains of Δ*Aoatg8* and Δ*Aoatg15* (data not shown), supporting the notion that the results obtained in the deletion mutants were truly due to the lack of autophagy activity. Besides, increased formation of PAS-like structures in Δ*Aoypt7* and Δ*Aoatg15* in the non-starved condition could be due to the accumulation of those involved in autophagy-mediated homeostatic recycling because of the blockade of downstream processes, while in the wild type, these PAS-like structures quickly disappeared soon after their formation as autophagy proceeded.

In *S. cerevisiae*, selective autophagy of organelles is mediated by receptor proteins (selective autophagy receptors) which exist on the substrates to be degraded. For example, in mitophagy, Atg32 is located on the mitochondrial outer membrane [30], whereas in pexophagy and nucleophagy, Atg36 and Atg39 reside on the peroxisomal membrane [31] and nuclear membrane [8], respectively. The adapter protein Atg11 binds to these receptors and induces autophagosome formation by recruiting core autophagy proteins [32]. In the *A. oryzae* mutant deleted for *Aoatg11* (AO090003000718), an ortholog of *ATG11*, nucleophagy as well as mitophagy activities induced by nitrogen starvation were partially suppressed (unpublished data), suggesting that *A. oryzae* also has a nucleophagy receptor. Besides, the diameter of autophagic bodies in *S. cerevisiae* is less than 1 µm [33], whereas that accumulated in the vacuoles of Δ*Aoatg15* was larger than 1 µm (Fig. 4), suggesting that unusually large autophagosomes that engulf the whole nucleus are formed in *A. oryzae* and a mechanism that specifically recognizes nuclei exists.

Nucleophagy has also been reported in other filamentous fungi. In the infection cycle of rice blast fungus *Magnaporthe oryzae*, nuclear degeneration occurs during the development of appressorium, a specialized infection structure. In this process, conidial-derived nuclei are distributed to the appressorium through mitosis, but the conidia then undergo cell death as the nuclei are degraded by the macroautophagy mechanism [34]. Autophagy-deficient strains form appressoria but do not undergo conidial cell death, impair efficient invasion hyphae formation, and lose infectivity to rice [35]. In the soil-borne disease fungus *Fusarium oxysporum*, during the vegetative hyphal fusion, the nucleus in the invading hyphae replicates by mitosis and migrates to the invaded hyphae where the resident nucleus is degraded in an Atg8-dependent manner [36]. Nucleophagy in *A. oryzae* is distinct from these in that it is induced by nutrient starvation and occurs in the multinucleated cells, as opposed to mononucleated cells of *M. oryzae* and *F. oxysporum*, implying that the degradation of whole nuclei does not directly lead to cell death. Although the physiological significance of nucleophagy in *A. oryzae* is not clear, it is tempting to speculate whether it has a role in the recycling/resuse of DNA-derived cellular components, not only amino acids.

## Supplementary Information

Below is the link to the electronic supplementary material.

Supplementary file1 (PDF 588 KB)

## Abbreviations

HOPS: Homotypic vacuole fusion and protein sorting
INM: Inner nuclear membrane
ONM: Outer nuclear membrane
PAS: Pre-autophagosomal structure

## References

[1] Anding AL, Baehrecke EH (2017) Cleaning house: selective autophagy of organelles. Dev Cell 41:10–22 28399394 10.1016/j.devcel.2017.02.016 PMC5395098

[2] Knodler LA, Celli J (2011) Eating the strangers within: host control of intracellular bacteria via xenophagy. Cell Microbiol 13:1319–1327 21740500 10.1111/j.1462-5822.2011.01632.x PMC3158265

[3] Mizushima N, Komatsu M (2011) Autophagy: renovation of cells and tissues. Cell 147:728–741 22078875 10.1016/j.cell.2011.10.026

[4] Suzuki K, Kirisako T, Kamada Y, Mizushima N, Noda T, Ohsumi Y (2001) The pre-autophagosomal structure organized by concerted functions of *APG* genes is essential for autophagosome formation. EMBO J 20:5971–5981 11689437 10.1093/emboj/20.21.5971 PMC125692

[5] Feng Y, He D, Yao Z, Klionsky DJ (2014) The machinery of macroautophagy. Cell Res 24:24–41 24366339 10.1038/cr.2013.168 PMC3879710

[6] Park Y, Hayashi YK, Bonne G, Arimura T, Noguchi S, Nonaka I, Nishino I (2009) Autophagic degradation of nuclear components in mammalian cells. Autophagy 5:795–804 19550147 10.4161/auto.8901

[7] Mochida K, Nakatogawa H (2022) Atg39 binding to the inner nuclear membrane triggers nuclear envelope deformation in piecemeal macronucleophagy. Autophagy 18:3046–3047 35468041 10.1080/15548627.2022.2069957 PMC9673966

[8] Mochida K, Oikawa Y, Kimura Y, Kirisako H, Hirano H, Ohsumi Y, Nakatogawa H (2015) Receptor-mediated selective autophagy degrades the endoplasmic reticulum and the nucleus. Nature 522:359–362 26040717 10.1038/nature14506

[9] Lee C, Wilfling F, Ronchi P, Allegretti M, Mosalaganti S, Jentsch S, Beck M, Pfander B (2020) Selective autophagy degrades nuclear pore complexes. Nat Cell Biol 22:159–166 32029894 10.1038/s41556-019-0459-2

[10] Shoji J, Kikuma T, Arioka M, Kitamoto K (2010) Macroautophagy-Mediated Degradation of Whole Nuclei in the Filamentous Fungus *Aspergillus oryzae*. PLoS ONE 5:e15650 21187926 10.1371/journal.pone.0015650 PMC3004950

[11] Ohsumi Y, Nakatogawa H, Suzuki K, Kamada Y (2009) Dynamics and diversity in autophagy mechanisms: lessons from yeast. Nat Rev Mol Cell Biol 10:458–467 19491929 10.1038/nrm2708

[12] Bas L, Papinski D, Licheva M, Torggler R, Rohringer S, Schuschnig M, Kraft C (2018) Reconstitution reveals Ykt6 as the autophagosomal SNARE in autophagosome-vacuole fusion. J Cell Biol 217:3656–3669 30097514 10.1083/jcb.201804028 PMC6168255

[13] Kriegenburg F, Bas L, Gao J, Ungermann C, Kraft C (2019) The multi-functional SNARE protein Ykt6 in autophagosomal fusion processes. Cell Cycle 18:639–651 30836834 10.1080/15384101.2019.1580488 PMC6464585

[14] Gao J, Reggiori F, Ungermann C (2018) A novel in vitro assay reveals SNARE topology and the role of Ykt6 in autophagosome fusion with vacuoles. J Cell Biol 217:3670–3682 30097515 10.1083/jcb.201804039 PMC6168247

[15] Watanabe Y, Iwasaki Y, Sasaki K, Motono C, Imai K, Suzuki K (2023) Atg15 is a vacuolar phospholipase that disintegrates organelle membranes. Cell Rep 42:113567 38118441 10.1016/j.celrep.2023.113567

[16] Kagohashi Y, Sasaki M, May AI, Kawamata T, Ohsumi Y (2023) The mechanism of Atg15-mediated membrane disruption in autophagy. J Cell Biol 222:e202306120 37917025 10.1083/jcb.202306120 PMC10622257

[17] Machida M, Asai K, Sano M, Tanaka T, Kumagai T, Terai G, Kusumoto K, Arima T, Akita O, Kashiwagi Y, Yu J, Bhatnagar D, Cleveland TE (2005) Genome sequencing and analysis of *Aspergillus oryzae*. Nature 438:1157–1161 16372010 10.1038/nature04300

[18] Takahashi T, Masuda T, Koyama Y (2006) Identification and analysis of Ku70 and Ku80 homologs in the koji molds *Aspergillus sojae* and *Aspergillus oryzae*. Biosci Biotechnol Biochem 70:135–143 16428831 10.1271/bbb.70.135

[19] Higuchi Y, Arioka M, Kitamoto K (2009) Endocytic recycling at the tip region in the filamentous fungus *Aspergillus oryzae*. Commun Integr Biol 8:327–328 10.4161/cib.2.4.8385 PMC2734037 19721880

[20] Kikuma T, Ohneda M, Arioka M, Kitamoto K (2006) Functional analysis of the *ATG8* homologue *Aoatg8* and role of autophagy in differentiation and germination in *Aspergillus oryzae*. Eukaryot Cell 5:1328–1336 16896216 10.1128/EC.00024-06 PMC1539149

[21] Maruyama J, Nakajima H, Kitamoto K (2001) Visualization of Nuclei in *Aspergillus oryzae* with EGFP and Analysis of the Number of Nuclei in Each Conidium by FACS. Biosci Biotechnol Biochem 65:1504–1510 11515532 10.1271/bbb.65.1504

[22] Yanagisawa S, Kikuma T, Kitamoto K (2013) Functional analysis of *Aoatg1* and detection of the Cvt pathway in *Aspergillus oryzae*. FEMS Microbiol Lett 338:168–176 23136971 10.1111/1574-6968.12047

[23] Kikuma T, Kitamoto K (2011) Analysis of autophagy in *Aspergillus oryzae* by disruption of *Aoatg13*, *Aoatg4*, and *Aoatg15* genes. FEMS Microbiol Lett 316:61–69 21204928 10.1111/j.1574-6968.2010.02192.x

[24] Nair U, Thumm M, Klionsky DJ, Krick R (2011) GFP-Atg8 protease protection as a tool to monitor autophagosome biogenesis. Autophagy 7:1546–1550 22108003 10.4161/auto.7.12.18424 PMC3327617

[25] Nishio J, Takahashi Y, Kasahara M, Takeda Y, Kikuma T (2023) AeiA is a novel autophagy-related protein that promotes peroxisome degradation by pexophagy in *Aspergillus oryzae*. FEBS Lett 597:608–617 36700830 10.1002/1873-3468.14589

[26] Kikuma T, Mitani T, Kohara T, Maruyama J, Kitamoto K (2017) Carbon and nitrogen depletion-induced nucleophagy and selective autophagic sequestration of a whole nucleus in multinucleate cells of the filamentous fungus *Aspergillus oryzae*. J Gen Appl Microbiol 63:139–146 28331162 10.2323/jgam.2016.09.001

[27] Tadokoro T, Kikuma T, Kitamoto K (2015) Functional analysis of AoAtg11 in selective autophagy in the filamentous fungus *Aspergillus oryzae*. Fungal Biol 119:560–567 26058532 10.1016/j.funbio.2015.03.001

[28] Suzuki K, Kubota Y, Sekito T, Ohsumi Y (2007) Hierarchy of Atg proteins in pre-autophagosomal structure organization. Genes Cells 12:209–218 17295840 10.1111/j.1365-2443.2007.01050.x

[29] Nguyen LN, Bormann J, Le GTT, Stärkel C, Olsson S, Nosanchuk JD, Giese H, Schäfer W (2011) Autophagy-related lipase FgATG15 of *Fusarium graminearum* is important for lipid turnover and plant infection. Fungal Genet Biol 48:217–224 21094265 10.1016/j.fgb.2010.11.004

[30] Kanki T, Wang K, Cao Y, Baba M, Klionsky DJ (2009) Atg32 is a mitochondrial protein that confers selectivity during mitophagy. Dev Cell 17:98–109 19619495 10.1016/j.devcel.2009.06.014 PMC2746076

[31] Motley AM, Nuttall JM, Hettema EH (2012) Pex3-anchored Atg36 tags peroxisomes for degradation in *Saccharomyces cerevisiae*. EMBO J 31:2852–2868 22643220 10.1038/emboj.2012.151 PMC3395097

[32] Nakatogawa H (2020) Autophagic degradation of the endoplasmic reticulum. Proc Jpn Acad 96:1–9 10.2183/pjab.96.001 PMC6974406 31932525

[33] Takeshige K, Baba M, Tsuboi S, Noda T, Ohsumi Y (1992) Autophagy in yeast demonstrated with proteinase-deficient mutants and conditions for Its induction. J Cell Biol 119:301–311 1400575 10.1083/jcb.119.2.301 PMC2289660

[34] He M, Kershaw MJ, Soanes DM, Xia Y, Talbot NJ (2012) Infection-associated nuclear degeneration in the rice blast fungus *Magnaporthe oryzae* requires non-selective macro-autophagy. PLoS ONE 7:e33270 22448240 10.1371/journal.pone.0033270 PMC3308974

[35] Veneault-Fourrey C, Barooah M, Egan M, Wakley G, Talbot NJ (2006) Autophagic fungal cell death is necessary for infection by the rice blast fungus. Science 312:169–174 10.1126/science.1124550 16645096

[36] Corral-Ramos C, Roca MG, Di Pietro A, Roncero MIG, Ruiz-Roldán C (2015) Autophagy contributes to regulation of nuclear dynamics during vegetative growth and hyphal fusion in *Fusarium oxysporum*. Autophagy 11:131–144 25560310 10.4161/15548627.2014.994413 PMC4507430

