# Supplementary material for: Nucleophagy in Aspergillus oryzae is Mediated by Autophagosome Formation and Vacuole-Mediated Degradation
Source: Curr Microbiol. 2024 Aug 20;81(10):315. doi: 10.1007/s00284-024-03838-y (PMC11335778; doi:10.1007/s00284-024-03838-y)
Supplement: Supplementary file 1 — Supplementary file1 (PDF 588 KB) [file 284_2024_3838_MOESM1_ESM.pdf]

Supplementary

Strain name	Genotype	Reference
RIB40	Wild-type	Machida et al., 2005
NSRku70-1-1	$niaD^{-}$ sC^{-} $adeA^{-}$ Δ*argB* Δ*ku70*::*argB*	Escaño et al., 2009
NSRku70-1-1A	$niaD^{-}$ sC^{-} $adeA^{-}$ Δ*argB* Δ*ku70*::*argB* *adeA*	Escaño et al., 2009
PA8GAtg8	$niaD^{-}$ sC^{-} $adeA^{-}$ Δ*argB* Δ*ku70*::*argB* Δ*Aoatg8*::*adeA* *PAoatg8-egfp-Aoatg8*::*niaD*	Tadokoro et al., 2015
NSRku70-1-1A AnH2B-EGFP	$niaD^{-}$ sC^{-} $adeA^{-}$ Δ*argB* Δ*ku70*::*argB* *adeA* *Ph2b-h2b-egfp*::*niaD*	This study
Δ*Aoatg1*	$niaD^{-}$ sC^{-} $adeA^{-}$ Δ*argB* Δ*ku70*::*argB* Δ*Aoatg1*::*adeA*	Yanagisawa et al., 2013
ΔA1EA8	$niaD^{-}$ sC^{-} $adeA^{-}$ Δ*argB* Δ*ku70*::*argB* Δ*Aoatg1*::*adeA* *PAoatg8-egfp-Aoatg8*::*niaD*	Yanagisawa et al., 2013
DAoatg1AnH2B	$niaD^{-}$ sC^{-} $adeA^{-}$ Δ*argB* Δ*ku70*::*argB* Δ*Aoatg1*::*adeA* *Ph2b-h2b-egfp*::*niaD*	This study
ΔAoatg8-1-1	$niaD^{-}$ sC^{-} $adeA^{-}$ Δ*argB* Δ*ku70*::*argB* Δ*Aoatg8*::*adeA*	Kikuma et al., 2006
DAoatg8AnH2B	$niaD^{-}$ sC^{-} $adeA^{-}$ Δ*argB* Δ*ku70*::*argB* Δ*Aoatg8*::*adeA* *Ph2b-h2b-egfp*::*niaD*	This study
Δ*Aoatg15*	$niaD^{-}$ sC^{-} $adeA^{-}$ Δ*argB* Δ*ku70*::*argB* Δ*Aoatg15*::*adeA*	Kikuma et al., 2011
DAoatg15AoAtg8	$niaD^{-}$ sC^{-} $adeA^{-}$ Δ*argB* Δ*ku70*::*argB* Δ*Aoatg15*::*adeA* *PAoatg8-egfp-Aoatg8*::*niaD*	This study
DAoatg15AnH2B	$niaD^{-}$ sC^{-} $adeA^{-}$ Δ*argB* Δ*ku70*::*argB* Δ*Aoatg15*::*adeA* *Ph2b-h2b-egfp*::*niaD*	This study
DAoypt7	$niaD^{-}$ sC^{-} $adeA^{-}$ Δ*argB* Δ*ku70*::*argB* Δ*Aoypt7*::*adeA*	This study
DAoypt7AoAtg8	$niaD^{-}$ sC^{-} $adeA^{-}$ Δ*argB* Δ*ku70*::*argB* Δ*Aoypt7*::*adeA* *PAoatg8-egfp-Aoatg8*::*niaD*	This study
DAoypt7AnH2B	$niaD^{-}$ sC^{-} $adeA^{-}$ Δ*argB* Δ*ku70*::*argB* Δ*Aoypt7*::*adeA* *Ph2b-h2b-egfp*::*niaD*	This study

Table S1 Strain list

Primer name	Sequence
pUC19_Aoypt7_up_Fw	CTCGGTACCCGGGGATCGCTGCGGCGTGAGTCCTGTGA
pUC19_Aoypt7_up_Rv	GTCTAGCACCCATGCGGCCGGCTGTGGCTATGTAAAGA
pUC19_Aoypt7_down_Fw	AGCTCGGTACCCGGGGATCGCTAGCTACCCTAGTGATGGACATG
pUC19_Aoypt7_down_Rv	AGGTCGACTCTAGAGGATCATCGTCTGGGACGTAGGTC
pUC19_adeA_up	GCTCGGTACCCGGGGATCGCGGCCGCATGGGTGCTAGACTCACAT
pUC19_adeA_down	CCATCACTAGGGTAGCTAGCTAGACCGCAGGAACCTTA
Aoypt7 check 500-F	TCCTCTGTCCTCTCCTGAGA
Aoypt7 check 500-R	TACGGTCGTGTATTGCAGGC

Table S2 Primer list

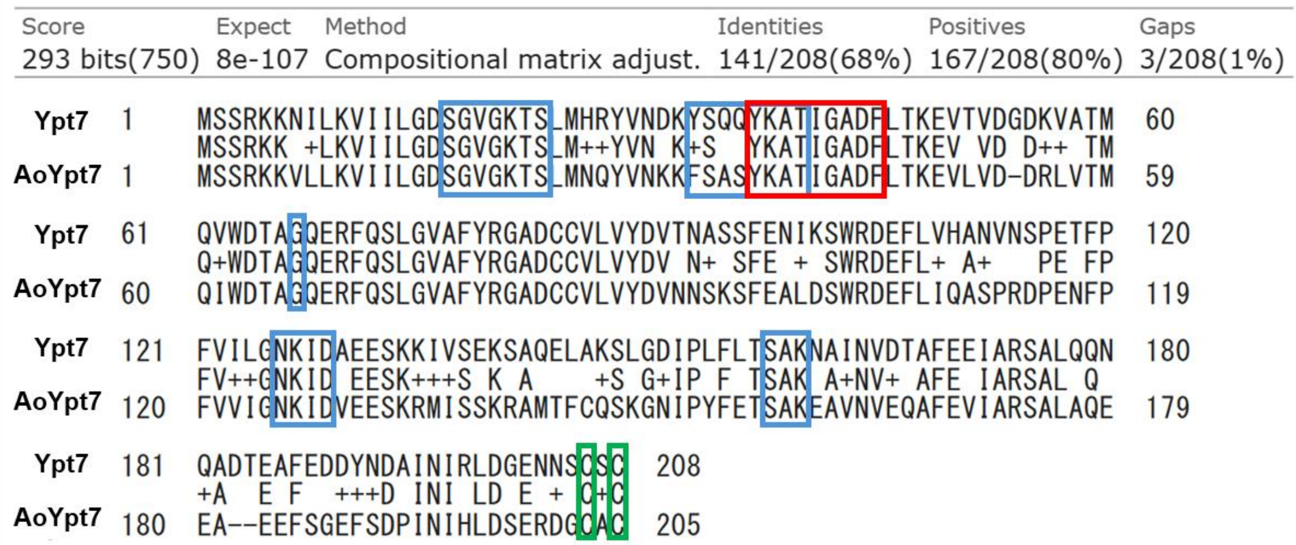

Fig. S1 Amino acid sequence alignment of Ypt7 and AoYpt7

Amino acid sequence alignment of Ypt7 (P32939) from *S. cerevisiae* and AoYpt7 (XP_001824054) from *A. oryzae* showing 68% identity. Red, blue, and green boxes indicate the binding region to the HOPS complex, the GTP binding regions, and the cysteine residues to be geranylgeranylated, respectively.

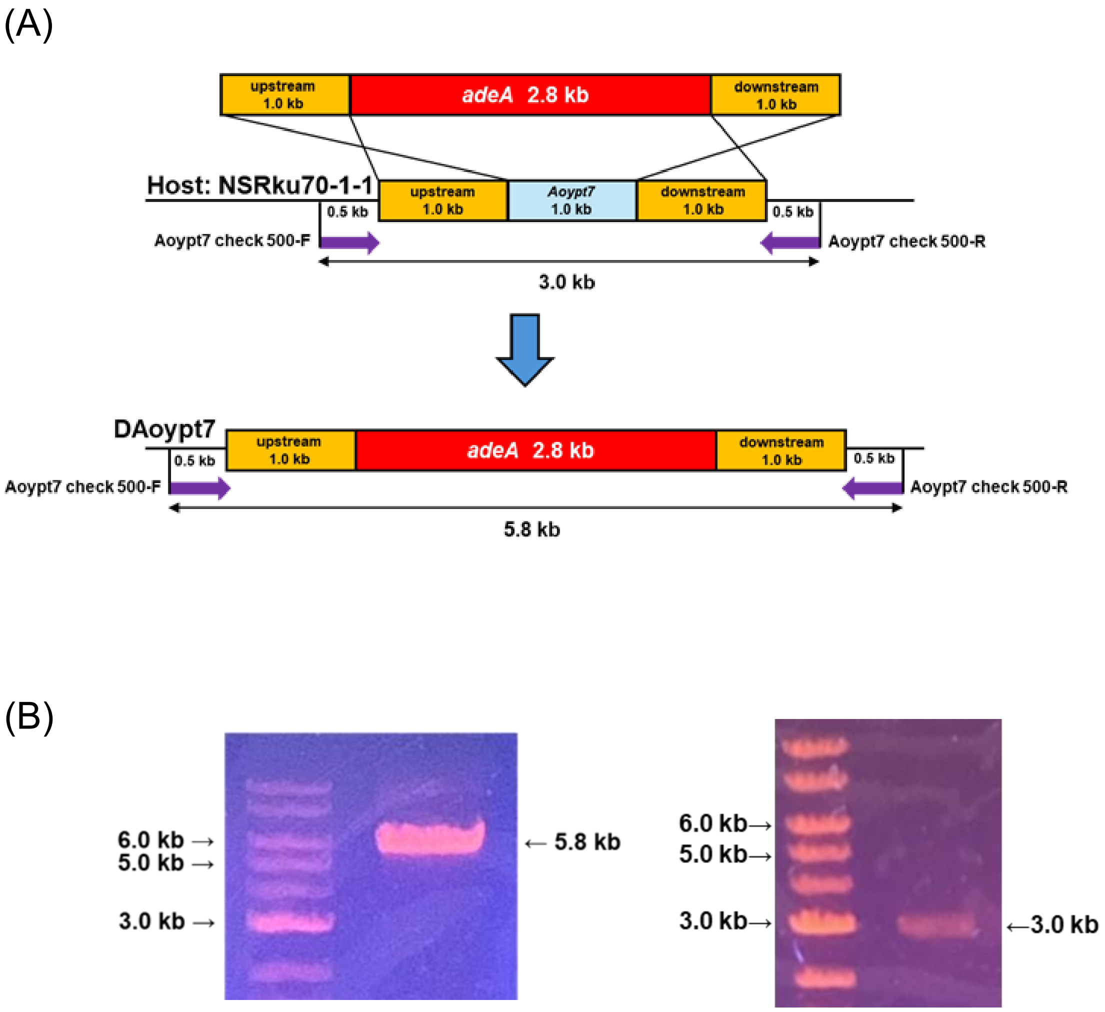

Fig. S2 Construction of *Aoypt7* disruptant

(A) *Aoypt7* gene of the host strain NSRku70-1-1 was disrupted by homologous recombination using the *adeA* selection marker. DNA fragment amplified with pUC19_Aoypt7_up_Fw and pUC19_Aoypt7_down_Rv primers using the plasmid pUC19_Aoypt7_deletion as a template was used for transformation.

(B) PCR amplification was performed using Aoypt7 check 500-F and Aoypt7 check 500-R as primers and the genomes recovered from the transformant (left) and NSRku70-1-1 (right), respectively, as templates. 5.8 kb band was correctly obtained in the gene disruptant.

(A)

(B)

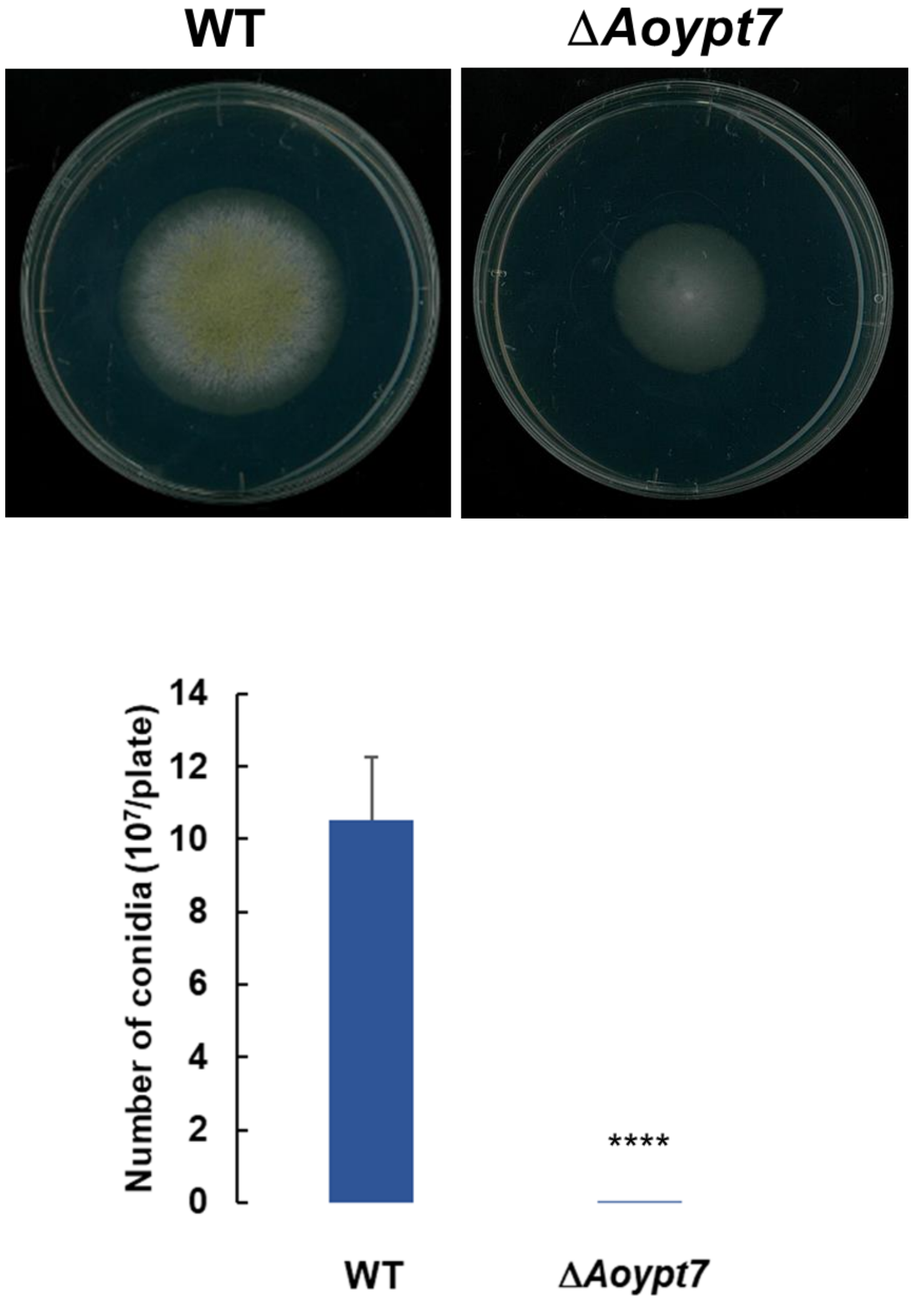

Fig. S3 Growth phenotypes of Δ*Aoypt7* strain

(A) Wild-type (NSRku70-1-1A) and Δ*Aoypt7* (DAoypt7) strains were inoculated onto PD plates and incubated for 4 d at 30°C. (B) Conidia were collected from the plates and the numbers were counted using a hemocytometer. Error bars, standard deviation (n=3). ****, p<0.0001 by t-test.
